# Student Initiative Improves HIV Screening Rate in Student-Run Free Clinic

**DOI:** 10.7759/cureus.5994

**Published:** 2019-10-25

**Authors:** Taylor Den Hartog, Laura Rezac, Chandler Jansen, Tej I Mehta, Cody Ness, Carol Whitman, Mark Beard

**Affiliations:** 1 Surgery, University of South Dakota Sanford School of Medicine, Sioux Falls, USA; 2 Dermatology, University of South Dakota Sanford School of Medicine, Sioux Falls, USA; 3 Radiology, University of South Dakota Sanford School of Medicine, Sioux Falls, USA; 4 Psychiatry, University of South Dakota Sanford School of Medicine, Sioux Falls, USA; 5 Family Medicine, University of South Dakota Sanford School of Medicine, Sioux Falls, USA

**Keywords:** hiv, quality improvement, student, rural medicine, infectious diseases, system initiative, free clinic, screening

## Abstract

Background

The Center for Disease Control provides recommendations for preventative services and screenings including recommendations for a one-time HIV screening of all adult patients between the ages of 13-64. But not all clinics are fully compliant with these recommendations. We identified a need for increased screening at two clinics in a rural setting. As a healthcare quality improvement initiative, we developed educational informatics to increase screening compliance.

Methods

This project assessed HIV screening rates before and after educational interventions at two clinics, the Coyote Clinic and the Avera Downtown Clinic. Three changes were implemented to increase the HIV screening rate and ultimately provide more effective high-quality health care. The three initiatives focused on patients, physicians, and student volunteers in order to provide a strong foundation of knowledge to all parties involved in a patient’s care.

Results

Prior to any interventions, the baseline screening rate (screenings/100 persons) at the Avera Downtown Clinic was 0.84 while the screening rate at the Coyote Clinic was 0.00. After the proposed interventions, the screening rate of the Downtown Clinic improved to 3.97 and the screening rate at the Coyote Clinic improved to 29.4. Using a Fisher’s Exact test, we found a statistically significant post-intervention increase in HIV screening at the Coyote Clinic after the intervention (p = 0.0002) but not at the Downtown Clinic (p = 0.0940.)

Conclusion

HIV screening rates improved after the implementation of interventional education initiatives tailored for patients, medical students, and physicians. Implementation of low-cost quality improvement measures such as the ones detailed herein may significantly improve long-term patient management, particularly in the context of screening tests.

## Introduction

Disease prevention and early detection are critical elements in providing high-quality health care. The Center for Disease Control (CDC) has released guidelines for disease prevention and screening for numerous diseases. One such disease is Human Immunodeficiency Virus (HIV). The CDC recommends that everyone between the ages of 13-64 should be screened at least once in an individual’s lifetime, and those with certain risk factors be screened once yearly [1]. As of 2015, it is estimated that, of the 1.1 million individuals with HIV in the United States, 15% did not know that they were infected [1]. Many of these individuals may unknowingly transmit the virus to others [2]. Moreover, in 2017, 38,739 people in the United States were diagnosed with HIV, with 7.5% of the new diagnoses happening in the Midwest [3]. Ethnicities that had the highest rate of diagnosis were African Americans and Hispanics [3]. Notably, although African Americans comprise 12% of the US population, they accounted for 44% of new HIV diagnoses. Likewise, Hispanics comprise 18% of the US population and accounted for 25% of new HIV diagnoses [3]. Early HIV detection by screening is paramount for the initiation of appropriate antiviral therapies, reduction of HIV-related illness, and reduced transmission rates [1]. In this study, we sought to improve HIV screening practices at a rural clinic for underserved populations via a student-led screening initiative.

## Materials and methods

Participants

This project utilized two clinics with similar catchment areas: The Avera Medical Group Health Care Clinic (Avera Downtown Clinic) and the Coyote Clinic. The Avera Downtown Clinic is an internal medicine clinic in Sioux Falls, SD, that provides care to uninsured members of the community. The Coyote Clinic is a medical student-run free clinic supervised by attending physicians and provides basic healthcare and screening to uninsured community members aged 16 or older. A multidisciplinary care team identified low screening rates at both clinics and recognized that almost all patients seen at the two clinics qualified for HIV screening, prompting the study herein.

All the patients seen in the Coyote Clinic from May 8, 2018 to November 27, 2018 and all patients seen at the Avera Downtown Clinic between July 31, 2018 and September 24th, 2018 were initially included in this study. Patient inclusion criteria were: age 13-64 with no previous HIV screening or HIV symptomatology. Patients requesting HIV screening due to believed exposure were excluded from this study.

Interventions

On August 28, 2018, several changes were implemented at the Coyote Clinic to increase the HIV screening rate. Interventions focused on patients, physicians, and medical student volunteers, respectively.

*Patient Interventions: *Patient-focused interventions aimed to reduce the perceived stigma associated with HIV screening. This stigma contributes to the late diagnosis of HIV infection and interferes with equitable testing of all patients [4]. CDC informational guides were placed in each exam room and were offered in English and Spanish at both clinic sites.

*Physician Interventions: *Each physician at Coyote Clinic received a letter informing him or her of the HIV screening initiative. The letter provided information on HIV screening guidelines, informed them of the initiative, and encouraged them to increase HIV screening.

**Figure 1:**
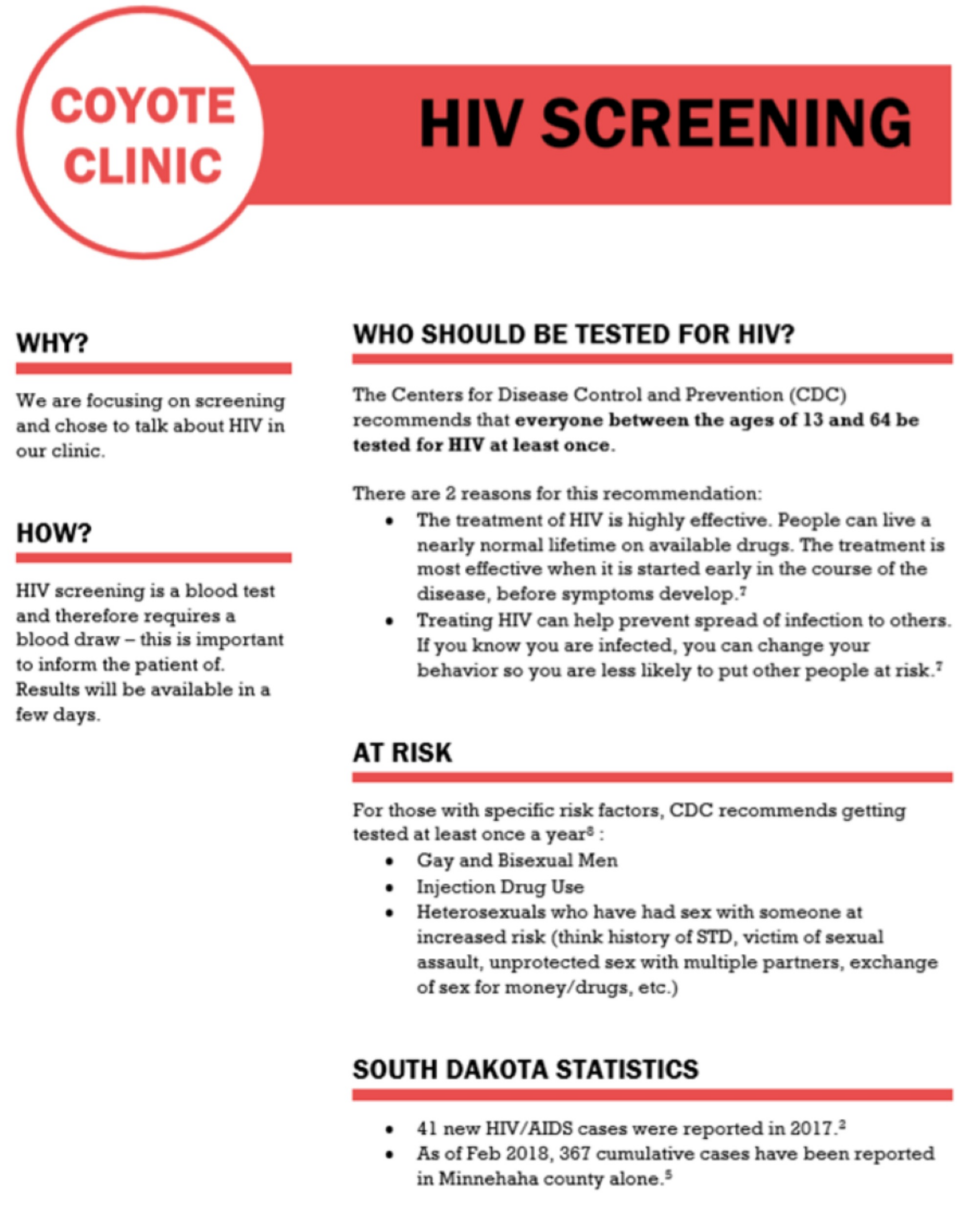
HIV screening handout presented verbally to student volunteers prior to every clinic day.

*Student Interventions:* Before each clinic day, students received verbal education on HIV screening and received a handout created for this project that includes information on HIV screening guidelines, risk factors, local HIV diagnosis statistics, and best practice language to use with patients (Figure 1). 

 

Additionally, an example statement of how to initiate the conversation about HIV screening was given. This proposed example statement was as follows: 

“Guidelines for HIV screening recommend one-time screening for everybody between the ages of 13 and 64, therefore we would like to screen you for HIV today unless you would like to decline. Do you have any questions?”

The example statement utilized ‘opt-out’ language. Opt-out screening entails that the provider will be screening for HIV after notifying the patient, but also that the patient may elect to decline or defer testing. Assent is inferred unless the patient declines testing [5]. This is in comparison to opt-in consent methods in which patients are offered testing and assent is required [6]. The opt-out method is thought to be less stigmatizing than using opt-in consent methods as well as more effective as rates of HIV screening are consistently higher at settings that use opt-out screening [5-6].

Statistical analysis

HIV screening incidence was calculated as the total number of patients screened over the total number of patients eligible to be screened. The screening incidence between and among each clinic, pre- and post-intervention was assessed using Fischer’s Exact test.

## Results

HIV screening rates pre- and post-intervention at both clinics are illustrated in Table 1. The Avera Downtown Clinic provided care for 118 eligible patients pre-intervention with a baseline screening rate of 0.84 screenings/100 persons. Post-intervention, 151 eligible patients were seen with a screening rate of 3.97 screenings/100 persons. Screening incidence pre- and post-intervention at the Avera Downtown Clinic was not statistically significant (p = 0.0940).

**Table 1:** Number of patients seen and the number of eligible patients screened at each clinic pre- and post-intervention.

	Avera Downtown Clinic Pre-Intervention	Avera Downtown Clinic Post-Intervention	Coyote Clinic Pre-Intervention	Coyote Clinic Post-Intervention
Patients Seen	205	221	56	54
Symptomatic	13	5	5	2
Outside Age Recommendation	26	21	2	1
Previously Screened	48	44	8	17
Eligible	118	151	41	34
Screened	1	6	0	10
Screening Rate (per 100 persons)	0.84	3.97	0	29.4

The Coyote Clinic provided care to 41 eligible patients pre-intervention. The baseline screening rate for the Coyote Clinic was 0 screenings/100 persons. Post-intervention 34 eligible patients were seen with a screening rate of 29.4 screenings/100 persons. This difference was statistically significant (p = 0.0002). Of the patients seen at Coyote Clinic, 48.7% identified as White/Caucasian, 26.1% Hispanic, 18.3% Black/African American, and 7% American Indian/Alaskan Native.

The screening incidences between Coyote Clinic and the Avera Downtown Clinic were not significantly different pre-intervention, but the screening incidence at Coyote Clinic was significantly greater than the Avera Downtown Clinic post-intervention (p < 0.001).

## Discussion

The goal of this project was to increase HIV screening incidence at two free clinics in rural settings via patient, physician, and student interventions. The student-led clinic, Coyote Clinic, saw a significantly greater, seven-fold increase in HIV screening incidence post-intervention. The physician-led clinic, Avera Downtown Clinic, also increased screening incidence post-intervention; however, this difference was not statistically significant.

Screening incidence between both clinics’ pre-intervention was not significantly different. Potential reasons for the low pre-intervention screening incidence in both groups may include physician and patient ignorance of current CDC guidelines for routine HIV screening, lack of routine screening habits, and patients unwilling to discuss risk-factors and patient-perceived stigma surrounding HIV. We postulate that the significant difference between both clinics post-intervention was due to the verbal education intervention, which only occurred at Coyote Clinic. This may illustrate the importance of framing effects to daily practice and our evidence suggests that providing pre-clinic medical education on a regular basis may encourage providers to offer appropriate screening to future patients.

While the use of opt-out screening language for HIV screening may have significantly contributed to the increased HIV screening rate, the posters with HIV screening information did not appear to have a significant effect on screening incidence. If the posters with CDC guidelines had a significant effect, it would be expected that the screening rate would have increased at both the Avera Downtown Clinic and Coyote Clinic; however, this was not the case.

This study highlights the utility of low-cost informational practice improvements, particularly in low-resource settings. With daily practice updates, in the age of electronic medical systems, primary care physicians are burdened by shifting guidelines. General practitioners care for diverse patient populations necessitating broad socio-medical edification for appropriate management. Low-cost, quality improvement measures, such as the ones detailed herein, patently improve patient management. Moreover, multidisciplinary leadership may better identify areas for quality improvement measures.

We identified various study limitations that could be mitigated through certain methodologies. For example, this study was conducted in two clinics with similar demographics, reducing the external validity of these results. This study could be conducted in clinics with different demographics to better understand the impact of demography on these quality improvement measures. Additionally, future studies could be conducted in various specialty clinics to identify the effects of specialty training on these measures. Finally, assessment of specific barriers to HIV screening could allow for more targeted methodologies and improve screening.

## Conclusions

We implemented patient, medical student, and physician quality improvement edification for HIV screening and noted a concomitant increase in HIV screening rates at a student-run free clinic. Verbal education immediately prior to the start of the clinic day appeared to be the most effective measure for HIV screening. In addition to education, opt-out HIV screening language may have significantly contributed to enhancing screening rates. Implementation of low-cost quality improvement measures such as the ones detailed herein may significantly improve long-term patient management, particularly in the context of screening tests.
